# Thermodynamic and Kinetic Aspects of Calcium Oxalate Crystallization and Renal Lithiasis

**DOI:** 10.3390/biom15081141

**Published:** 2025-08-07

**Authors:** Jaume Dietrich, Antònia Costa-Bauza, Félix Grases

**Affiliations:** 1 Renal Lithiasis and Pathological Calcification Group, Research Institute of Health Sciences (IUNICS), University of the Balearic Islands, 07122 Palma, Spain; jaume.dietrich@uib.cat (J.D.); fgrases@uib.es (F.G.); 2 Health Research Institute of the Balearic Islands (IdISBa), 07010 Palma, Spain

**Keywords:** calcium oxalate, crystallization, thermodynamics effects, kinetic effects, hydroxycitrate, phytate

## Abstract

Thermodynamic factors (supersaturation of substances that form crystals) and kinetic factors (heterogeneous nucleants and crystallization inhibitors) affect the formation of crystals and stones in the urinary tract. We studied the effect of five different polyhydroxycarboxylic acids and phytate on the formation of calcium oxalate crystals in artificial urine. All tested molecules are known to inhibit the crystallization of this calcium salt, and to also form complexes with calcium ions. Considering the typical concentration of polyhydroxycarboxylic acids in urine (similar to that of the calcium ion) and their ability to inhibit crystallization, their most important effect is the capacity to complex calcium—a thermodynamic effect. For phytate and its metabolites, which are present in concentrations much lower than that of the calcium ion, the most important effect is as a crystallization inhibitor—a kinetic effect. Among the five polyhydroxycarboxylic acids examined here, hydroxycitrate had the strongest complexing capacity, and the addition of phytate to hydroxycitrate led to greater inhibition of crystallization. Therefore, because oral consumption of hydroxycitrate does not increase the urinary pH, it is likely that the combined consumption of hydroxycitrate and phytate can provide certain benefits for patients with increased risk of developing calcium oxalate stones. We also discussed the effects of these different molecules on the different calcium oxalate stones, including papillary calcium oxalate monohydrate stones, cavity calcium oxalate monohydrate stones, calcium oxalate dihydrate stones, and mixed calcium oxalate dihydrate/hydroxyapatite stones.

## 1. Introduction

Studies by Fleisch [1,2] in the 1970s demonstrated that three fundamental factors must be considered to explain the formation of kidney stones: the urinary concentration (supersaturation) of stone-forming molecules, the effect of molecules that promote crystallization, and the effect of molecules that inhibit crystallization. Research on the kinetics of crystallization inhibitors (molecules that inhibit nucleation, growth, and/or aggregation) showed that this activity occurs after adsorption of the inhibitor on the nuclei or faces of the crystal during development. For this reason, the effects of an inhibitor may be greater at low supersaturation than at high supersaturation. This is because the type of nucleation and crystal growth depend on the extent of supersaturation, in that these processes are nonspecific and very strong at high supersaturation; therefore, inhibitors are less effective at high supersaturation [3,4]. Nucleation is the key step in crystal formation. Homogeneous nucleation, which only involves crystallizing molecules, is slow and only occurs at high supersaturation [5,6]. Heterogeneous nucleation, which is induced by preformed solid particles, is more rapid although it depends on the nature of the different molecular constituents [5,6]. Most kidney stones result from heterogeneous nucleation [7,8].

The thermodynamic effects of inhibitors are related to their interactions with one of the substances that generate the solid forming complexes, clusters, new substances, etc. A consequence of this interaction is a decrease in supersaturation, and this decreases the driving force of crystallization and the amount of the molecule that can crystallize, so there is an apparent increase in its solubility. This is a thermodynamic effect that is independent of time. Kinetic and thermodynamic effects are both affected by interactions of an inhibitor with one of the species that forms crystals, even if it is already part of the crystal lattice. For this reason (as described below), some inhibitors can affect the kinetics and thermodynamics of crystal formation. Typically, the thermodynamic effects on calcium oxalate crystallization require high concentration of the substance that produces these effects (of the same order as that of urinary calcium), since by forming stable complexes in solution, supersaturation decreases significantly. The kinetic effects usually occur at low concentrations of the inhibitor relative to those of the crystallizing compound, as in the case of phytate, and these effects are more noticeable at moderate supersaturations.

The role of some proteins and other biological macromolecules (e.g., glycosaminoglycans) in calcium-oxalate nephrolithiasis is more complex. While some authors attribute crystallization-inhibiting properties to them [9], others consider that they play a promoting role, facilitating heterogeneous aggregation and nucleation [10,11]. Although both processes have been observed in in vitro laboratory studies, the in vivo observations are probably not so easy to explain. For example, the secretion of fibronectin can be stimulated by calcium oxalate crystals, and this protein, excreted by the renal tubular cells, can prevent calcium oxalate crystals from binding to them [12], thus preventing the formation of plugs that would ultimately induce the development of papillary calcium oxalate monohydrate stones [13]. Therefore, although the action of these biological macromolecules may be related to oxalocalcic calculogenesis, their participation would be indirect in many cases. Another clear example is a recent study showing that the protein sclerostin regulates renal calcium excretion, and the deletion of the sclerostin gene in mice significantly diminishes urinary calcium excretion and increases calcium reabsorption [14]. Obviously, these changes affect calcium oxalate supersaturation (a thermodynamic factor).

Currently, many plant extracts with potential antilithiasic properties have been described. Many of these studies are based on traditional medicine. Although some of these studies demonstrate a certain inhibitory capacity of these extracts on calcium oxalate crystallization, the most notable effects of most of them are related to their antioxidant properties [15,16], demonstrating a possible important role in preventing the development of oxidative stress lesions in the renal papilla, which are often the cause of papillary calcium oxalate monohydrate stones [13]. In some cases, it has also been shown that certain extracts can reduce the endogenous synthesis of oxalate [17], which indirectly leads to a decrease in calcium oxalate supersaturation (thermodynamic factor).

Hydroxycitrate is an effective inhibitor of calcium oxalate crystallization [18,19,20], and this effect is explained by two important properties. On the one hand, hydroxycitrate can form stable complexes with soluble calcium ions [21]. The formation of these complexes decreases the concentration of free calcium in the medium, so supersaturation—the thermodynamic driving force of crystallization—decreases and this leads to decreased precipitation and an apparent increase in solubility. On the other hand, hydroxycitrate also interacts with crystals that have already formed, and this hinders the processes of nucleation and crystallization and leads to a decreased crystallization rate—a kinetic effect. In this sense, the effects of hydroxycitrate are equivalent to those of citrate, a product widely used for treatment of oxalocalcic nephrolithiasis [22]. Comparisons of the kinetic and thermodynamic effects of citrate and hydroxycitrate have generally concluded that hydroxycitrate has a stronger effect. An important difference between these products is that citrate is partially metabolized after ingestion and this can increase the urinary pH above 7 [22,23]; hydroxycitrate is generally not metabolized so its consumption does not alter urinary pH [18,19,20]. The concentrations of citrate and hydroxycitrate normally found in urine are in the order of 0.5–2 mM [18,19,20,21].

Phytate (inositol hexaphosphate, InsP6) is one of the most potent inhibitors of the formation and development of calcium oxalate crystals, in that it inhibits nucleation and crystal growth processes [24]. The in vivo effects of phytate are also attributable to the body’s generation of dephosphorylated metabolites (InsP5, InsP4, InsP3, InsP2), which also inhibit the crystallization of calcium oxalate [25]. Although phytate forms complexes with calcium, the urinary concentration achieved following consumption is very low, so the decrease in supersaturation caused by a decrease in urinary free calcium is not significant. The efficacy of phytate and its dephosphorylation products as inhibitors of calcium oxalate crystallization can be explained by the strong interaction of the phosphate groups with calcium of crystals surface and the structure of the phytate molecule, which effectively blocks the growth of calcium oxalate crystals at different steps. Importantly, the ingestion of phytate does not alter urinary pH. The concentrations of phytate normally found in urine are in the order of 0.5–2 µM [25].

In this study, we evaluated the kinetic and thermodynamic effects of five different polyhydroxycarboxylic acids and phytate, together and separately, on the crystallization of calcium oxalate, and analyzed the effects of these inhibitors on the formation of different types of calcium oxalate stones. The main objective of this paper is to evaluate the effectiveness of combining a substance such as hydroxycitrate (which does not affect urinary pH) that has clear thermodynamic effects on the crystallization of calcium oxalate (reduction in supersaturation), with a product such as phytate, which exhibits clear kinetic effects on this crystallization.

## 2. Materials and Methods

### 2.1. Reagents and Solutions

Hydroxycitrate (hydroxycitric acid tripotassium salt monohydrate) was obtained from Toronto Research Chemicals Inc. (Toronto, ON, Canada). Citrate (sodium citrate tribasic dihydrate), tartronate (tartronic acid), tartrate (L-tartaric acid disodium salt dihydrate), malonate (malonic acid), and phytate (phytic acid sodium salt hydrate) were obtained from Sigma-Aldrich (St. Louis, MO, USA). Synthetic urine components and sodium oxalate were obtained from Panreac (Montcada i Reixac, Barcelona, Spain).

Synthetic urine was prepared by mixing equal volumes of solution A and solution B (Table 1) immediately before an experiment. These two solutions were prepared in ultra-pure deionized water from a Milli-Q system (Merck-Millipore, Darmstadt, Germany), filtrated, sonicated, and adjusted to pH 6.

After dissolution of the salts in solution A, but before sonication and adjustment of pH, 10 mL of a 1 M CaCl_2_ solution was carefully added to prevent the precipitation of calcium salts.

A total of 10 mM stock solutions of hydroxycitrate, citrate, tartrate, tartronate and malonate were prepared in solution B, and adjusted to pH 6 if necessary. In total, 1 mM stock solution of phytate was prepared in ultra-pure deionized water. After preparation, all solutions were stored at room temperature until use.

### 2.2. Crystallization Procedure

The effects of hydroxycitrate, citrate, tartrate, tartronate, malonate and phytate (see Table 2) on calcium oxalate crystallization in synthetic urine (pH 6) were determined using a previously described kinetic turbidimetric assay [25]. This method employed a spectrometer equipped with a fiber-optic light-guide measuring cell (AvaSpec-ULS2048CL-EVO, Avantes, Apeldoorm, The Netherlands) that was operated in the kinetic mode and integrated absorbance from 400 to 600 nm. These experiments were performed with constant mixing by a magnetic stir bar (250 rpm) at room temperature (25 °C) (for an initial screening) and in a water bath at 37 °C (to reproduce physiological conditions). pH was monitored to confirm that it remained stable. These conditions led to crystallization times similar to the residence time of urine in the human urinary tract. For experiments without additions, 25 mL of solution B was first added into a crystallization flask, and absorbance was continuously measured immediately after addition of 25 mL of solution A to create the artificial urine. This was followed by the addition of 0.705 mL of 40 mM sodium oxalate after 30 s to induce supersaturation, which triggers the crystallization of calcium oxalate. The time when absorbance first increased was considered to be the induction time of crystallization (t_i_).

To evaluate the effect of a molecule on the t_i_ of calcium oxalate, 25 mL of 10 mM polyhydroxycarboxylic acid stock solution prepared in solution B were mixed with 25 mL of solution A to obtain a final concentration of 5 mM; the final concentration of phytate was 2 µM because it is a stronger inhibitor and its physiological concentration is lower and it was achieved by adding 100 µL of 1 mM phytate stock solution to solution B. For compounds that increased the t_i_ of calcium oxalate, lower concentrations were used for experiments conducted at 37 °C to mimic physiological conditions. The desired concentrations were achieved by mixing the appropriate volumes of solution B and of 10 mM polyhydroxycarboxylic acid stock solution prepared in solution B. Mixtures of two inhibitors were also used to evaluate possible interactions.

### 2.3. Analysis of Crystal Structure and Morphology

The crystals formed during this assay were collected by passing the solution through a 0.45 µm pore nylon membrane filter and drying at room temperature. The crystals were fixed with an adhesive conductive tape onto a sample holder and then observed by scanning electron microscopy (SEM; TM4000 Plus II, Hitachi, Tokyo, Japan).

Collected crystals were also analyzed via powder X-Ray diffraction (PXRD, D8 Advance, Bruker, Berlin, Germany) and Fourier-Transform Infrared Spectroscopy (FT-IR, Tensor 27, Bruker, Berlin, Germany) analysis to identify the crystallized products and their different phases.

### 2.4. Calcium Complexation Procedure

Studies of calcium complexation by polyhydroxycarboxylic acids were performed using a calcium selective electrode. Calcium solutions of 0.1, 0.5, 1, 5, 10, 50, 100 and 500 mM of CaCl_2_ were prepared in 0.15 M NaCl. A calcium selective electrode (DX240-Ca; Mettler Toledo, Columbus, OH, USA) and a potentiometer (micropH 2002; Crisson, Barcelona, Spain) were used for measurements of electrical potential (mV). These values were transformed to log[Ca^2+^] for linear regression. Three modified versions of Solution A (Table 1) were prepared to obtain final concentrations of 2.5, 5, and 10 mM Ca^2+^. The effect of polyhydroxycarboxylic acids concentration (1, 2, and 4 mM) was assessed by mixing 5, 10, or 20 mL of the 10 mM stock solution with an appropriate volume of solution B to complete 25 mL, and 25 mL of each of the three modified versions of solution A, with a final volume of 50 mL. The concentration of free calcium in each assay was quantified by interpolation from a linear regression.

### 2.5. Statistics

The data are expressed as the mean of three replicates ± standard deviation. Two-sided ANOVA with Tukey’s post hoc test was performed to assess significancy on synergistic assays. All data analysis and interpretation were carried out using GraphPad Prism version 8.0.2 (GraphPad Software, La Jolla, CA, USA).

## 3. Results

### 3.1. Crystallization

**Figure 1:**
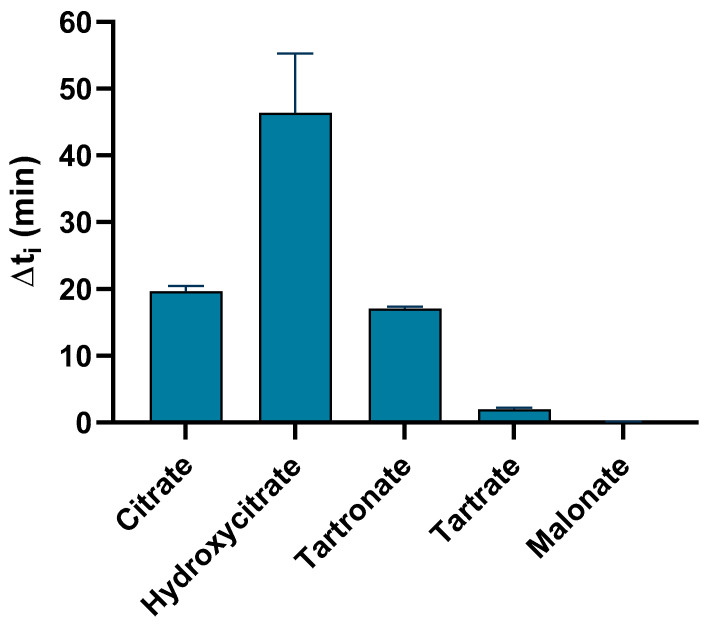
The effect of different polyhydroxycarboxylic acids (5 mM) on Δt_i_ in artificial urine at 25 °C and pH 6.0. Here and below: the initial oxalate concentration was 0.705 mM, and the initial calcium concentration was 200 mg/L; Δt_i_ = t_i_(inhibitor) − t_i_(control); the values indicate means ± SDs of three replicates.

We initially assessed the effect of five different polyhydroxycarboxylic acids and phytate on the t_i_ of calcium oxalate crystallization at room temperature (25 °C) in artificial urine. The control solution did not have an inhibitor, and the t_i_(control) was 3.6 ± 0.7 min. This value was reproducible and within the residence time of urine in the human urinary calices. The different polyhydroxycarboxylic acids had different t_i_(inhibitor) values relative to the control, and Δt_i_ values (t_i_[inhibitor] − t_i_[control]) are presented below (Figure 1).

Three of the five polyhydroxycarboxylic acids had strong inhibitory effects, but tartaric acid and malonic acid had very weak effects. Hydroxycitrate (∆t_i_ = 46.4 min) had the strongest effect, followed by citrate (∆t_i_ = 19.7 min) and tartronate (∆t_i_ = 17.1 min).

**Figure 2:**
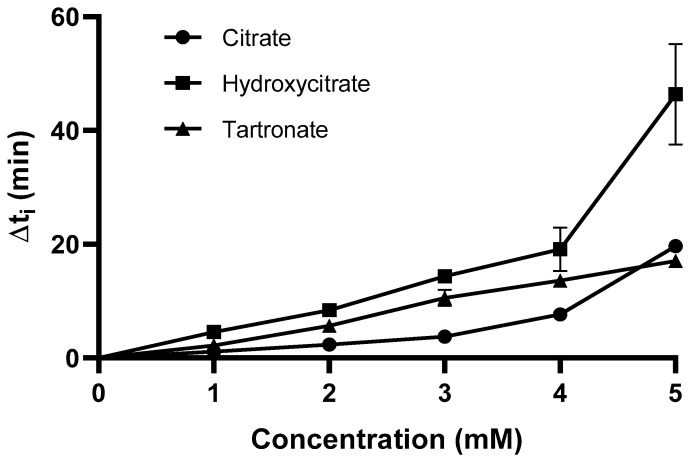
The effect of citrate, hydroxycitrate, and tartronate concentration on Δt_i_ in artificial urine at 25 °C and pH 6.0.

We also evaluated the effects of different concentrations of three polyhydroxycarboxylic acids on Δt_i_ (Figure 2). For each inhibitor, the Δt_i_ increased gradually with concentration up to 4 mM. The Δt_i_ had a large increase when the hydroxycitrate concentration increased from 4 mM (19.15 min ± 3.8) to 5 mM (46.40 ± 8.9), but this did not occur for citrate or tartronate.

**Figure 3:**
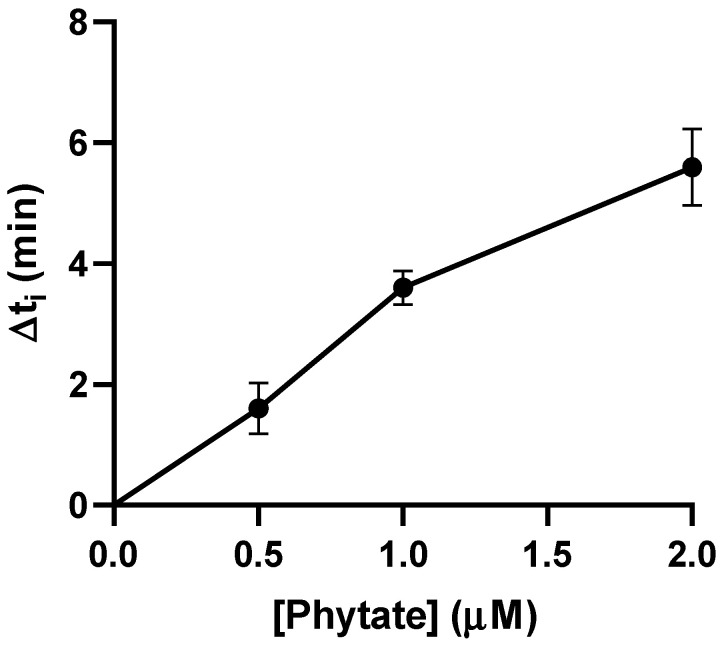
The effect of phytate concentration on Δti in artificial urine at 25 °C and pH 6.0.

Phytate is a well-known inhibitor of calcium oxalate crystallization, so we examined the effect of phytate concentration on Δt_i_ in our model system (Figure 3). The ∆ti was 5.6 min at a phytate concentration of 2 µM. Notably, this concentration of phytate is 2500-times lower than the maximum tested concentration of the polyhydroxycarboxylic acids (5 mM).

**Figure 4:**
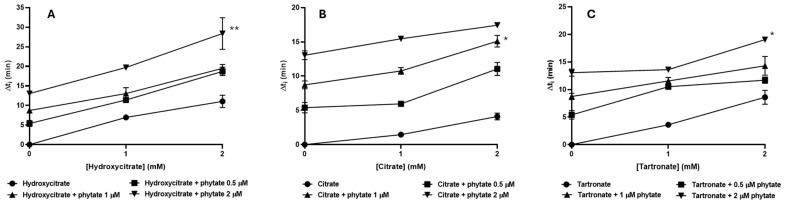
The effect of the concentration of hydroxycitrate (**A**), citrate (**B**), and tartronate (**C**) with different concentrations of phytate (0, 0.5, 1, 2 µM) on Δt_i_ in artificial urine at 37 °C and pH 6.0. Two-sided ANOVA test with a Tukey’s post hoc test was conducted to evaluate significant differences between each experimental induction time and the theoretical calculated by the sum of the two individual induction times. * *p* < 0.05; ** *p* < 0.005.

We further examined the effects of the three polyhydroxycarboxylic acids at concentrations of 0 to 2 mM in combination with phytate at concentrations of 0 to 2 µM on the Δt_i_ at 37 °C and pH 6 (Figure 4). These concentrations were selected because they allowed observations of potential interactions (because the Δt_i_ values were not too short) and they provided reproducible results (because the Δt_i_ values were not too long).

As expected, the results for individual polyhydroxycarboxylic acids at 37 °C show that the ∆t_i_ increased with the concentration of each molecule (Figure 4). Moreover, the addition of increasing concentrations of phytate further increased these ∆t_i_ values. The greater Δt_i_ values upon combination of individual polyhydroxycarboxylic acids with phytate suggests these effects may be synergistic or additive.

The mixture of hydroxycitrate with phytate led to a longer Δt_i_ than expected based on the sum of the induction times for each individual inhibitor at the same concentration. In particular, at the maximum concentrations tested (2 mM hydroxycitrate, 2 µM phytate), the ∆t_i_ (28.4 min) was greater than the sum of the Δt_i_ values of phytate alone (13 min) and hydroxycitrate alone (11 min) at the same concentrations, thus demonstrating a possible synergistic effect. Citrate and phytate mixtures also appeared to have a slightly synergistic interaction at concentrations 0.5 and 1 µM of phytate, in that the Δt_i_ values were about 1 min greater than expected from the sum of the Δt_i_ values of the individual molecules; however, this effect was very small and did not occur at a higher phytate concentration, arguing against synergism. The results of tartronate with phytate also showed no evidence of synergism.

### 3.2. Crystal Structure and Morphology

**Figure 5:**
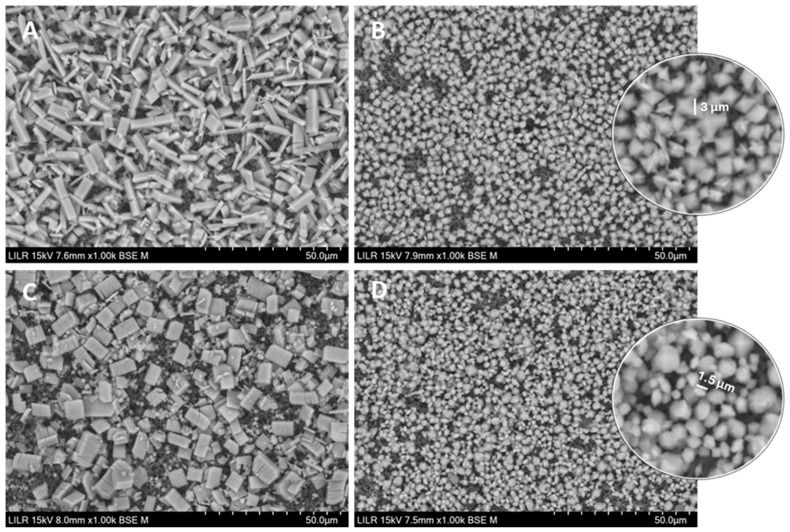
SEM of calcium oxalate crystals isolated after kinetic–turbidimetric assays in artificial urine at 37 °C in the control, without an inhibitor (**A**), with 2 µM phytate (**B**), with 2 mM hydroxycitrate (**C**), and with 2 mM hydroxycitrate + 2 µM phytate (**D**).

We used SEM to examine the morphology of crystals that formed without an inhibitor and the effects of hydroxycitrate and phytate (Figure 5). In the absence of an inhibitor (Figure 5A), the crystals were mainly calcium oxalate trihydrate (COT) and had dimensions of 9–12 µm × 3 µm, aprox. In the presence of 2 µM phytate alone (Figure 5B), the crystals were mainly calcium oxalate dihydrate (COD), and about 3 µm per face. In the presence of 2 mM hydroxycitrate alone (Figure 5C), there were mainly COT, with the longest face being 9 to 13 µm. In the presence of 2 mM hydroxycitrate and 2 µM phytate (Figure 5D), the crystals were mainly COD, small and round, with a morphology considerably different than conventional COD crystals as the obtained from crystallization in presence of 2 µM phytate (Figure 5B), with diameters of about 2–3 µm for the big ones and 1–1.5 µm for the small round crystals.

**Figure 6:**
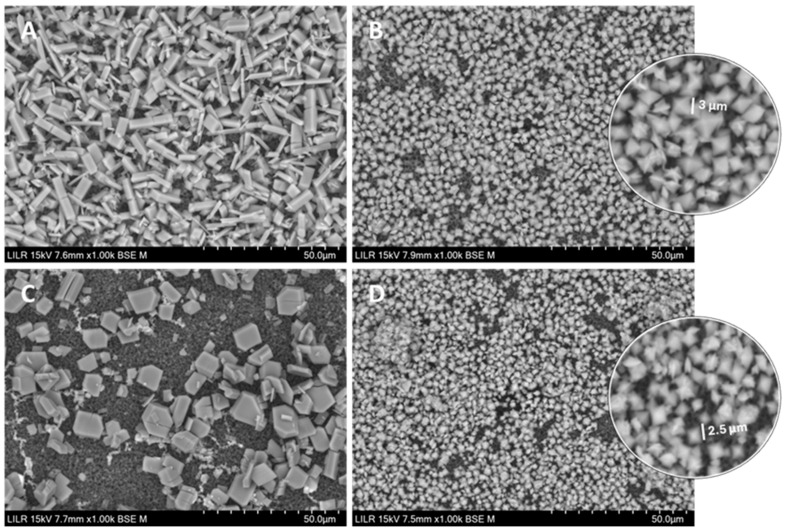
SEM of calcium oxalate crystals isolated after kinetic–turbidimetric assays in artificial urine at 37 °C in the control, without an inhibitor (**A**), with 2 µM phytate (**B**), with 2 mM citrate (**C**), and with 2 mM citrate + 2 µM phytate (**D**).

We used SEM to determine the effects of citrate and phytate on crystal morphology (Figure 6). In the absence of an inhibitor (Figure 6A), crystal morphology was the same as in Figure 5A. In the presence of 2 µM phytate alone (Figure 6B), the crystals were mainly COD, octahedral, and about 3 µm per face. In the presence of 2 mM citrate alone (Figure 6C), the crystals were COT, with the longest face of 10–15 µm. In the presence of 2 mM citrate and 2 µM phytate (Figure 6D) the crystals were COD with about 2.5 µm per face.

**Figure 7:**
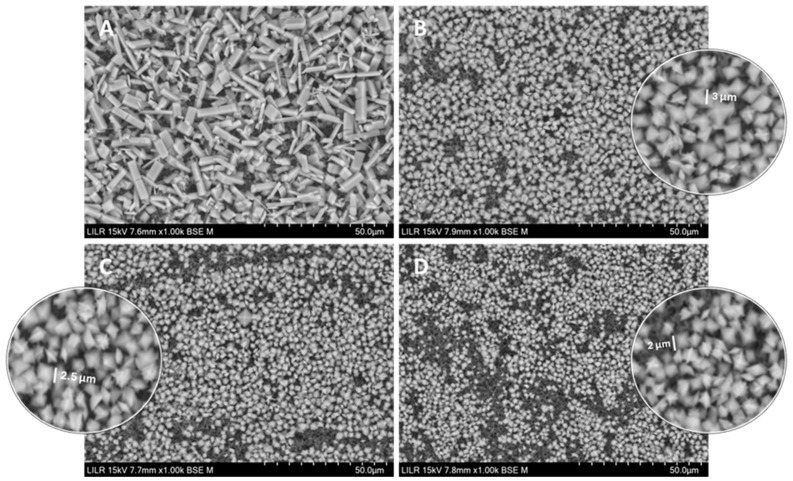
SEM of calcium oxalate crystals isolated after kinetic–turbidimetric assays in artificial urine at 37 °C in the control, without an inhibitor (**A**), with 2 µM phytate (**B**), with 2 mM tartronate (**C**), and with 2 mM tartronate + 2 µM phytate (**D**).

We used SEM to determine the effects of tartronate and phytate on crystal morphology (Figure 7). In the absence of an inhibitor (Figure 7A), crystal morphology was the same as in Figure 6A and Figure 5A. Calcium oxalate crystals obtained in the presence of 2 µM phytate (Figure 7B) were COD (~3 µm per face). Crystals obtained in the presence of 2 mM tartronate (Figure 7C) were COD with faces about 2.5 µm. In the presence of 2 mM tartronate and 2 µM phytate (Figure 7D), the crystals were COD with faces of about 2 µm.

FT-IR spectra of the collected crystals were obtained to corroborate the formation of calcium oxalate and the hydration form [26] (see Supporting Information).

**Figure 8:**
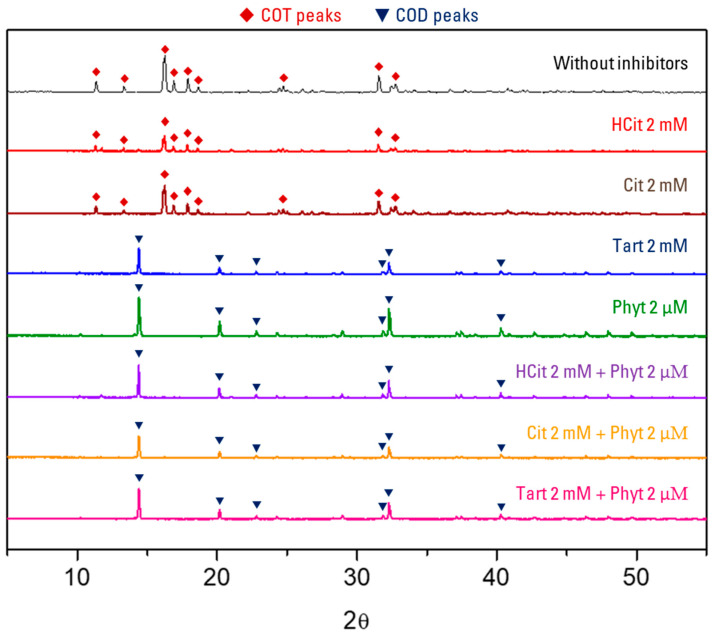
PXRD diffractograms of calcium oxalate crystals isolated after kinetic–turbidimetric assays in artificial urine at 37 °C in absence and presence of different tested compounds. The peaks have been related to the corresponding calcium oxalate hydrate [27].

PXRD diffractograms of the same collected crystals were also obtained to determine the crystal phase and composition of the crystals of each experiment. Two phases of calcium oxalate were identified: calcium oxalate trihydrate was present in the crystals obtained from experiments without inhibitors and in the presence hydroxycitrate 2 mM and citrate 2 mM, while calcium oxalate dihydrate was identified in the remaining experiments [27] (Figure 8).

Results from SEM (Figure 5, Figure 6 and Figure 7), FT-IR (see Supporting Information) and PXRD (Figure 8) show concordance in the observed calcium oxalate phases isolated from the different kinetic–turbidimetric assays, obtaining COT crystals for the experiments carried out in absence of inhibitor, and in presence of 2 mM of hydroxycitrate or 2 mM of citrate. COD crystals were obtained for the other assays.

### 3.3. Calcium Complexation

**Figure 9:**
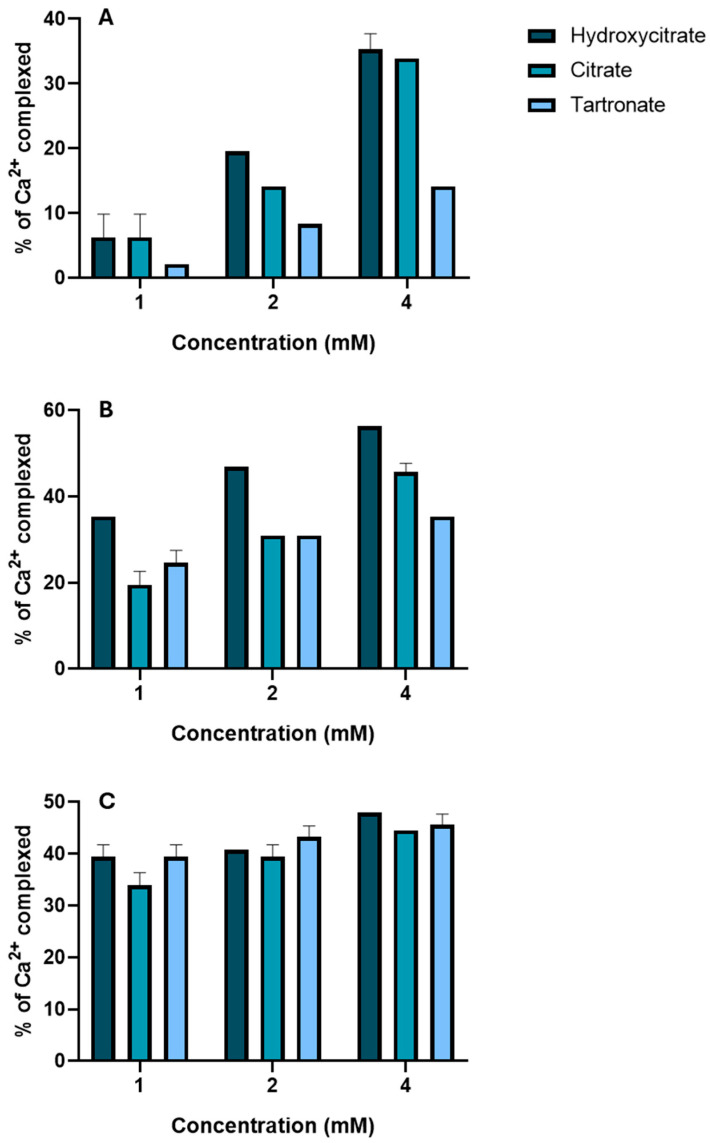
The effect of the concentration of hydroxycitrate, citrate, and tartronate (1, 2, or 4 mM), and a calcium concentration of 2.5 mM (**A**), 5 mM (**B**) and 10 mM (**C**) on calcium complexation.

We also evaluated the effect on calcium complexation of three different concentrations (1, 2, 4 mM) of the polyhydroxycarboxylic acids and at three different concentrations of calcium (2.5, 5.0, 10 mM) (Figure 9). The results show that a higher calcium concentration and a greater concentration of each polyhydroxycarboxylic acid led to a greater percentage of complexation. Moreover, the calcium complexation was greatest for hydroxycitrate, followed by citrate and then tartronate.

A hydroxycitrate concentration of 4 mM led to a calcium complexation of about 50% when the calcium concentration was 5 or 10 mM. These results are consistent with the crystallization studies, in that they demonstrate hydroxycitrate had the strongest inhibitory effect.

## 4. Discussion

There were three major results of this study. First, two inhibitors of calcium oxalate crystallization—hydroxycitrate and phytate—were effective at concentrations that are common in human urine, although we were unable to establish the nature of any possible interaction when both inhibitors were used together. Second, our studies of calcium complexation indicated that hydroxycitrate had the strongest effect, followed by citrate and tartronate. Third, the addition of crystallization inhibitors to artificial urine altered the morphology of the resulting calcium oxalate crystals, apparently due to changes in kinetics and thermodynamics. The crystalline morphology of calcium oxalate was affected to a greater degree by the mixture of 2 mM hydroxycitrate and 2 µM phytate (Figure 5D), observing COD crystals more round shaped and considerably smaller (1.5 µm per face) than conventional COD crystals as those obtained from the experiments with only 2 µM phytate (3 µm per face) (Figure 5B). This change in morphology, along with the increment of induction of crystallization times of the mixture of hydroxycitrate and phytate in comparison with the times obtained in presence of the inhibitors individually suggest a possible synergistic effect between hydroxycitrate and phytate.

### 4.1. Papillary COM Stones

Papillary calcium oxalate monohydrate (COM) stones typically form after a lesion in the intrapapillary tissue, a region with abundant collagen [28]. The interstitial fluid in this environment has a high pH (7.4), favoring the crystallization of hydroxyapatite. When passing through the cellular layer that covers the papilla and contacting the urine, COM can grow on this deposit. When the renal environment does not favor the formation of hydroxyapatite deposits, these types of stones are less likely. Therefore, inhibiting the development of intrapapillary calcifications can help prevent the formation of COM stones. The formation of hydroxyapatite begins with the formation of small clusters of calcium, hydroxide, and phosphate (Posner clusters), each with about ten units of calcium and phosphate [29]. This is facilitated by certain proteins that are grouped into larger ensembles, and intratissue calcifications develop if they are not eliminated. When molecules such as citrate, hydroxycitrate, pyrophosphate, bisphosphonates, and inositol phosphates bind to calcium, this inhibits the development of hydroxyapatite deposits [30]. Moreover, if hydroxyapatite occurs as colloidal particles in plasma or interstitial fluids, it can often be eliminated through the liver. The adsorption of citrate, hydroxycitrate, and inositol phosphates on these particles increases their negative surface charge, inhibits further aggregation, and facilitates elimination. Obviously, the elimination of these deposits by the immune system can also prevent tissue calcification [31]. When Randall’s plaque (a hydroxyapatite deposit) forms and comes into contact with urine, that is always supersaturated with calcium oxalate, as hydroxyapatite can facilitate the nucleation of calcium oxalate, this initiates the growth of COM crystals on hydroxyapatite; molecules that inhibit nucleation and/or crystal growth can delay the process but cannot prevent it. Therefore, to prevent the formation of this type of renal calculus, it is important to block ectopic calcification of the papillary tissue. Polyanions, such as citrate, hydroxycitrate, pyrophosphate, or inositol phosphates, are effective inhibitors of ectopic calcification, although in this case, these molecules generally do not act via a typical substrate–crystal interaction.

However, not all papillary stones are the same. Some of these stones form because of obstruction of the renal tubules due to a high calcium concentration and a high urinary pH. In turn, this induces the formation of intratubular calcium oxalate and hydroxyapatite crystals that cause lesions in the tubules and even lead to tubule obstruction. When part of the damaged and obstructed tubule is in constant contact with urine that is supersaturated with calcium oxalate, it will give rise to a typical COM concretion (sometimes with superficial COD crystals) or even to a COD stone attached to the papilla. The formation of these stones is usually linked to high supersaturation of calcium oxalate, so kinetic inhibitors of crystal development (nucleation and crystal growth) are usually not very effective. In this case, reducing calcium oxalate supersaturation is very important. Thus, the action of substances such as citrate and hydroxycitrate, which form complexes with calcium [20], are more important because they can significantly decrease supersaturation due to their complexation with calcium. However, citrate alkalinizes urine [18], and if the urinary pH exceeds 6.2, then hydroxyapatite crystallization will be favored and will generate stones by itself or induce the nucleation of calcium oxalate [20].

Hydroxycitrate is not readily metabolized and does not alter urinary pH [20]. Phytate and other inositol phosphates occur at concentrations much lower than urinary calcium, so even though they form complexes with calcium, their effect on calcium oxalate supersaturation is not significant. However, when there is high calciuria due to the excessive release of calcium from the bone, such as during osteoporosis or distal renal tubular acidosis, phytate and other inositol phosphates decrease the release of calcium from bone; this leads to decreased calciuria, decreased calcium oxalate supersaturation, and decreased calcium oxalate crystallization.

### 4.2. Non-Papillary COM Stones

Non-papillary COM stones usually develop in cavities that have low urodynamic efficiency, are usually spherical with one or more lobes, and have a central core. These stones typically develop in urine that has no significant abnormalities, and the origin of the stone (core) often has heterogeneous nucleants (uric acid, hydroxyapatite, organic matter, etc.). These nucleants undoubtedly induce stone formation, because urine is normally supersaturated with calcium oxalate. In this case, crystallization inhibitors may be effective because the supersaturation is not excessive; however, these inhibitors may be insufficient because the stone will continue to grow, although more slowly. In non-papillary COM stones, the presence of a heterogeneous nucleant is critical. Therefore, the most effective way to prevent their formation is to decrease the level of heterogeneous nucleants, for example, by raising the urinary pH above 5 when the stone core contains uric acid, or by lowering the urinary pH when the stone core contains hydroxyapatite. Inhibitors of heterogeneous nucleation can delay this process, but if a deposit with a heterogeneous nucleant has already formed, the stone will continue to develop.

### 4.3. COD Stones

COD stones are typically generated when the urine has a high calcium concentration (e.g., hypercalciuria), and molecules that inhibit nucleation and crystal growth will probably be less effective in this case. These stones often have small amounts of hydroxyapatite that acts as a heterogeneous nucleant. In this case, molecules that inhibit the heterogeneous nucleation of calcium oxalate (citrate, hydroxycitrate, inositol phosphates) can also be effective inhibitors. Undoubtedly, the most effective way to prevent development of COD stones is to decrease the concentration of free calcium in the urine. This can be achieved using complexing agents, such as citrate or hydroxycitrate, which inhibit the formation of COD crystals due to thermodynamic effects. However, citrate intake can increase the urinary pH above 6.2, which can then induce the accumulation of hydroxyapatite. As mentioned above, phytate and inositol phosphates can also decrease hypercalciuria when the calcium is released from bone.

### 4.4. Mixed COD/Hydroxyapatite Stones

Mixed COD/hydroxyapatite stones are generated when there is a high urinary calcium concentration (normally hypercalciuria) and the pH is above 6.2. This usually occurs in patients with primary hyperparathyroidism or distal renal tubular acidosis, and kinetic inhibitors will have little effect in these patients. Apart from lowering the urinary pH (when possible), decreasing the concentration of free urinary calcium (supersaturation of calcium oxalate) by use of calcium complexing agents (citrate and hydroxycitrate) can be effective. Furthermore, in the case of renal tubular acidosis, citrate can decrease systemic acidity without affecting urinary pH, which will remain high. In patients with distal renal tubular acidosis and osteoporosis, the excess urinary calcium comes from bone, and the use of phytate and inositol phosphates will be effective in decreasing calcium oxalate supersaturation [24].

## 5. Conclusions

Crystallization inhibitors act by binding to developing crystals during different stages of development, mainly at the nucleation step. This kinetic effect can be important when there is low supersaturation; to be effective, these inhibitors must stop crystal development until urine has left the kidneys, or at least the renal tubules and calyces. The combination of a crystallizing species with a new substance that gives rise to soluble products (through complexation, redox reactions, cluster formation, etc.) also leads to decreased supersaturation, which is the thermodynamic driving force of crystallization. It is important to consider that many molecules inhibit crystallization by both kinetic and thermodynamic mechanisms, in that they decrease supersaturation and disrupt nucleation and/or crystal growth.

For this reason, we suggest that the combination of hydroxycitrate and phytate may be particularly effective in decreasing the crystallization of calcium oxalate. Hydroxycitrate decreases supersaturation (a thermodynamic effect), and phytate inhibits nucleation and crystal growth (a kinetic effect). The main limitation of this study is that the results obtained correspond to in vitro studies and must be corroborated clinical studies in humans. Thus, clinical trials are needed to confirm the efficacy of this approach.

## Figures and Tables

**Table 1:** Composition of synthetic urine. Solutions A and B were filtrated, sonicated and adjusted to pH 6, and synthetic urine was obtained by mixing equal volumes of each solution.

Solution A			Solution B		
	Stock Solution (g/L)	Final Concentration (mM)		Stock Solution (g/L)	Final Concentration (mM)
Na_2_SO_4_·10H_2_O	6.23	19.34	NaH_2_PO_4_·2H_2_O	2.41	15.45
MgSO_4_·7H_2_O	1.46	5.92	Na_2_HPO_4_·12H_2_O	5.6	15.64
NH_4_Cl	4.64	86.75	NaCl	13.05	223.31
KCl	12.13	162.69			

**Table 2:** Polyhydroxycarboxylic acids and phytate evaluated in this study as inhibitors of the crystallization of calcium oxalate.

Complexing Agent	Chemical Structure	IUPAC Name (Acid Form)	Molar Mass (g·mol^−1^)	LD50 (mg/kg)	Natural Source
Hydroxycitrate	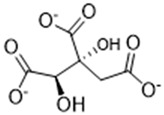	(1S,2S)-1,2-dihydroxypropate-1,2,3-tricarboxylic acid	208.12	>5000	*Garcinia cambogia*, *Garcinia indica*, *Hibiscus sabdariffa*
Citrate	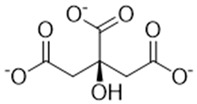	2-hydroxy-1,2,3-propanetricarboxylic acid	192.12	>5000	Citrus fruits, vegetables
Malonate	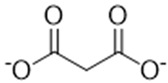	propanedioic acid	104.06	1310	Corn, Red beetroot, citrus fruits
Tartronate	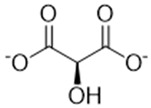	2-hydroxypropanedioic acid	120.06	Not documented	Cucurbit crops, various fruits
Tartrate	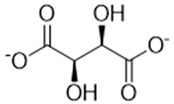	(2R,3R)-2,3-dihydroxybutanedioic acid	148.07	2660	Grapes, tamarind, bananas
Phytate	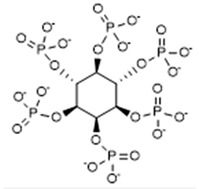	(1R,2R,3S,4S,5R,6S)-cyclohexane-1,2,3,4,5,6-hexayl hexakis-[dihydrogen(phosphate)]	660.04	>5000	Cereals, legunes, nuts, seeds

## Data Availability

The data that support the findings of this study are available from the corresponding author [A.C.-B.] upon reasonable request.

## Supplementary Materials

The following supporting information can be downloaded at https://www.mdpi.com/article/10.3390/biom15081141/s1: Figure S1.A: FT-IR spectrum of the crystals isolated from the assay of crystallization of calcium oxalate without inhibitors, corresponding to calcium oxalate trihydrate (COT); Figure S1.B: FT-IR spectrum of the crystals isolated from the assay of crystallization of calcium oxalate in the presence of 2 mM hydroxycitrate, corresponding to calcium oxalate trihydrate (COT); Figure S1.C: FT-IR spectrum of the crystals isolated from the assay of crystallization of calcium oxalate in the presence of 2 mM citrate, corresponding to calcium oxalate trihydrate (COT); Figure S1.D: FT-IR spectrum of the crystals isolated from the assay of crystallization of calcium oxalate in the presence of 2 mM tartronate, corresponding to calcium oxalate dihydrate (COD); Figure S1.E: FT-IR spectrum of the crystals isolated from the assay of crystallization of calcium oxalate in the presence of 2 µM phytate, corresponding to calcium oxalate dihydrate (COD); Figure S1.F: FT-IR spectrum of the crystals isolated from the assay of crystallization of calcium oxalate in the presence of 2 mM hydroxycitrate + 2 µM phytate, corresponding to calcium oxalate dihydrate (COD); Figure S1.G: FT-IR spectrum of the crystals isolated from the assay of crystallization of calcium oxalate in the presence of 2 mM citrate + 2 µM phytate, corresponding to calcium oxalate dihydrate (COD); Figure S1.H: FT-IR spectrum of the crystals isolated from the assay of crystallization of calcium oxalate in the presence of 2 mM tartronate + 2 µM phytate, corresponding to calcium oxalate dihydrate (COD).

## Abbreviations

The following abbreviations are used in this manuscript:

COM	Calcium oxalate monohydrate
COD	Calcium oxalate dihydrate
COT	Calcium oxalate trihydrate
