# Supplementary material for: Thermodynamic and Kinetic Aspects of Calcium Oxalate Crystallization and Renal Lithiasis
Source: Biomolecules. 2025 Aug 7;15(8):1141. doi: 10.3390/biom15081141 (PMC12384284; doi:10.3390/biom15081141)
Supplement: Supplementary file 1 [file biomolecules-15-01141-s001.zip › biomolecules-3754193-supplementary.pdf]

Supporting information

Thermodynamic and Kinetic Aspects of Calcium Oxalate Crystallization and Renal Lithiasis

Jaume Dietrich [1,2], Antònia Costa-Bauza [1,2,*] and Félix Grases [1,2]

[1] Renal Lithiasis and Pathological Calcification Group, Research Institute of Health Sciences (IUNICS), University of the Balearic Islands, 07122 Palma, Spain
[2] Health Research Institute of the Balearic Islands (IdISBa), 07010 Palma, Spain
***** Correspondence: antonia.costa@uib.es

Table of contents

Figure S1.A FT-IR spectrum of the crystals isolated from the assay of crystallization of calcium oxalate without inhibitors, corresponding to calcium oxalate trihydrate (COT). 3

Figure S1.B FT-IR spectrum of the crystals isolated from the assay of crystallization of calcium oxalate in the presence of 2 mM hydroxycitrate, corresponding to calcium oxalate trihydrate (COT)... 3

Figure S1.C FT-IR spectrum of the crystals isolated from the assay of crystallization of calcium oxalate in the presence of 2 mM citrate, corresponding to calcium oxalate trihydrate (COT). ... 4

Figure S1.D FT-IR spectrum of the crystals isolated from the assay of crystallization of calcium oxalate in the presence of 2 mM tartronate, corresponding to calcium oxalate dihydrate (COD). 4

Figure S1.E FT-IR spectrum of the crystals isolated from the assay of crystallization of calcium oxalate in the presence of 2 µM phytate, corresponding to calcium oxalate dihydrate (COD). .. 5

Figure S1.F FT-IR spectrum of the crystals isolated from the assay of crystallization of calcium oxalate in the presence of 2 mM hydroxycitrate + 2 µM phytate, corresponding to calcium oxalate dihydrate (COD)... 5

Figure S1.G FT-IR spectrum of the crystals isolated from the assay of crystallization of calcium oxalate in the presence of 2 mM citrate + 2 µM phytate, corresponding to calcium oxalate dihydrate (COD)... 6

Figure S1.H FT-IR spectrum of the crystals isolated from the assay of crystallization of calcium oxalate in the presence of 2 mM tartronate + 2 µM phytate, corresponding to calcium oxalate dihydrate (COD)... 6

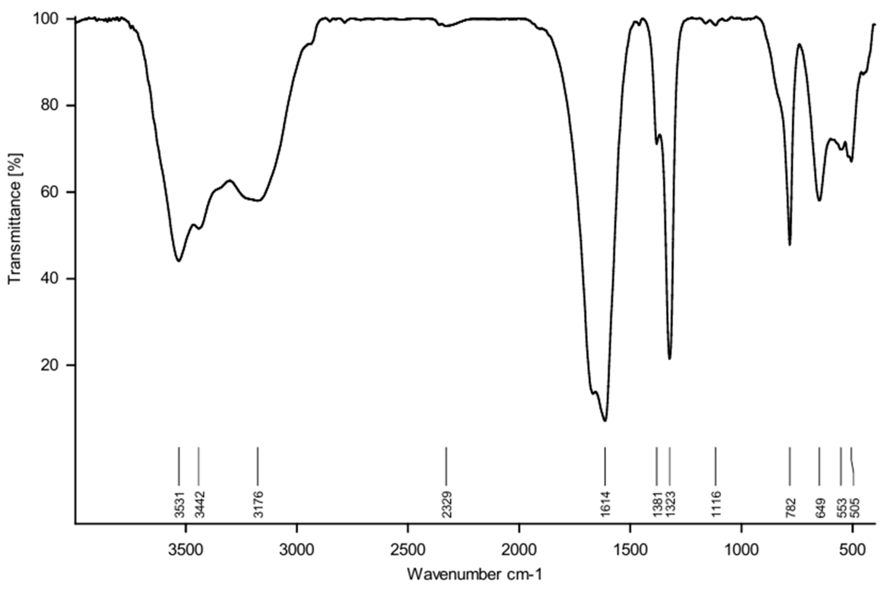

Figure S1.A FT-IR spectrum of the crystals isolated from the assay of crystallization of calcium oxalate without inhibitors, corresponding to calcium oxalate trihydrate (COT).

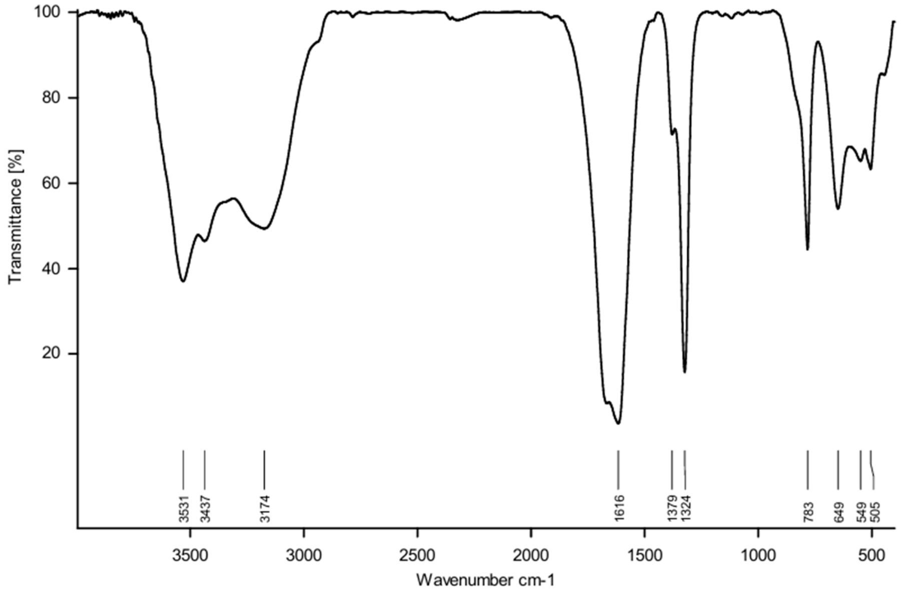

Figure S1.B FT-IR spectrum of the crystals isolated from the assay of crystallization of calcium oxalate in the presence of 2 mM hydroxycitrate, corresponding to calcium oxalate trihydrate (COT).

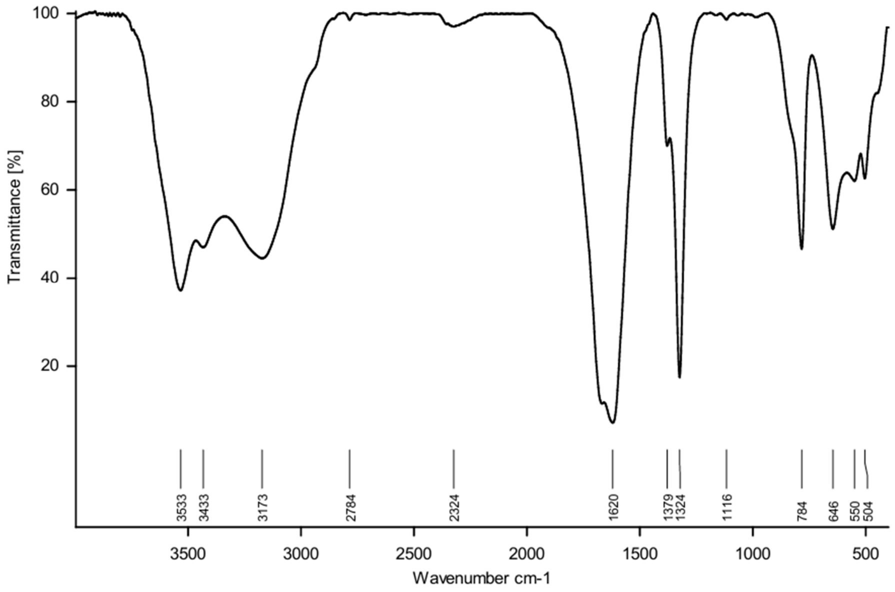

Figure S1.C FT-IR spectrum of the crystals isolated from the assay of crystallization of calcium oxalate in the presence of 2 mM citrate, corresponding to calcium oxalate trihydrate (COT).

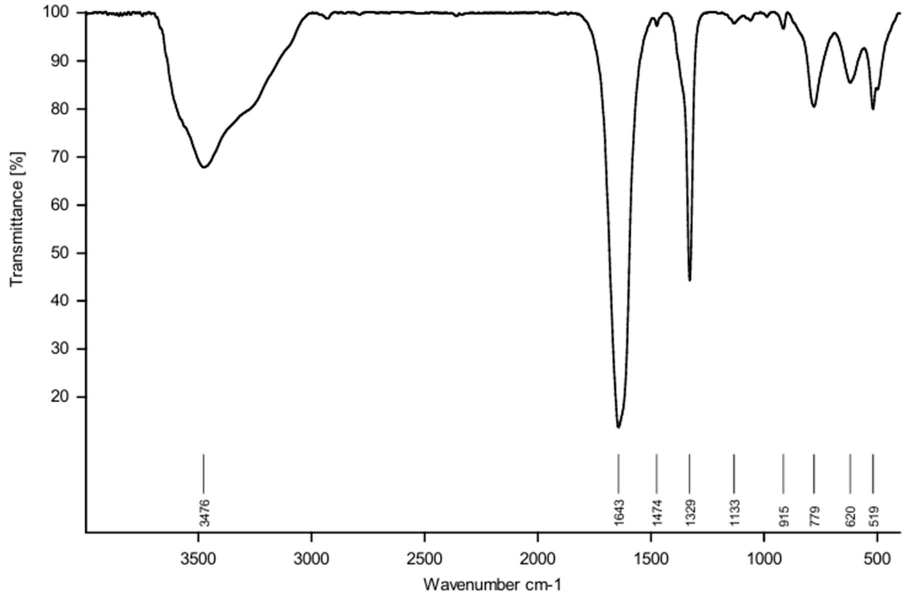

Figure S1.D FT-IR spectrum of the crystals isolated from the assay of crystallization of calcium oxalate in the presence of 2 mM tartronate, corresponding to calcium oxalate dihydrate (COD).

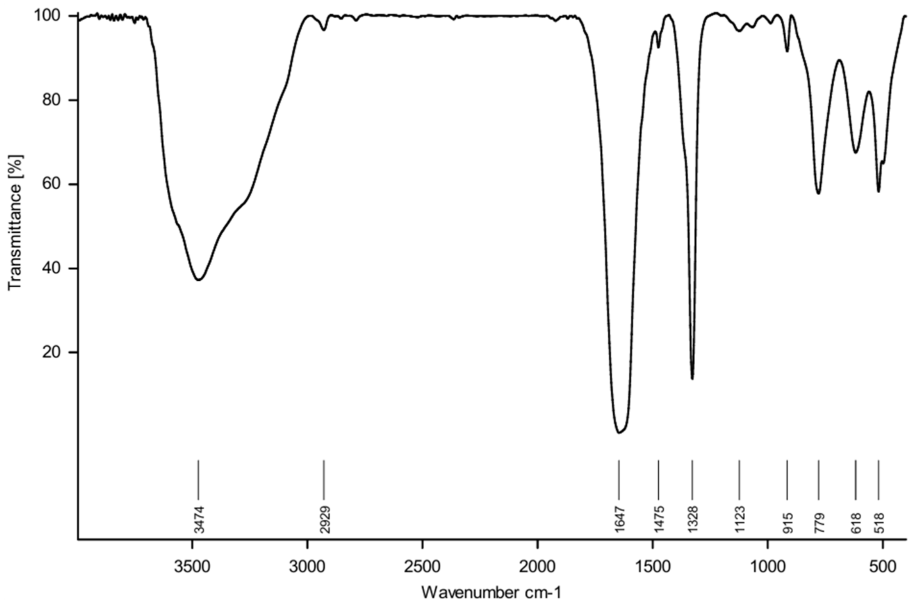

Figure S1.E FT-IR spectrum of the crystals isolated from the assay of crystallization of calcium oxalate in the presence of 2 µM phytate, corresponding to calcium oxalate dihydrate (COD).

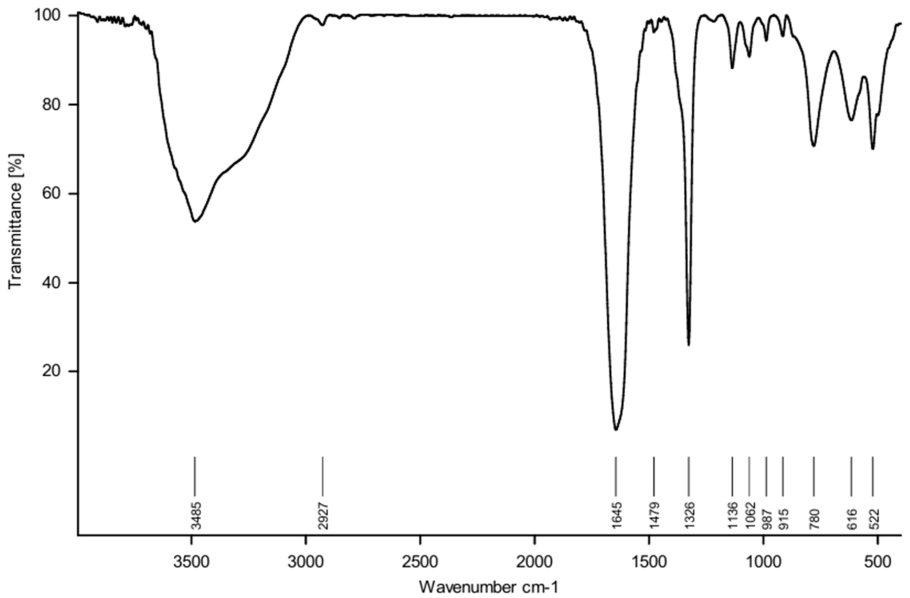

Figure S1.F FT-IR spectrum of the crystals isolated from the assay of crystallization of calcium oxalate in the presence of 2 mM hydroxycitrate + 2 µM phytate, corresponding to calcium oxalate dihydrate (COD).

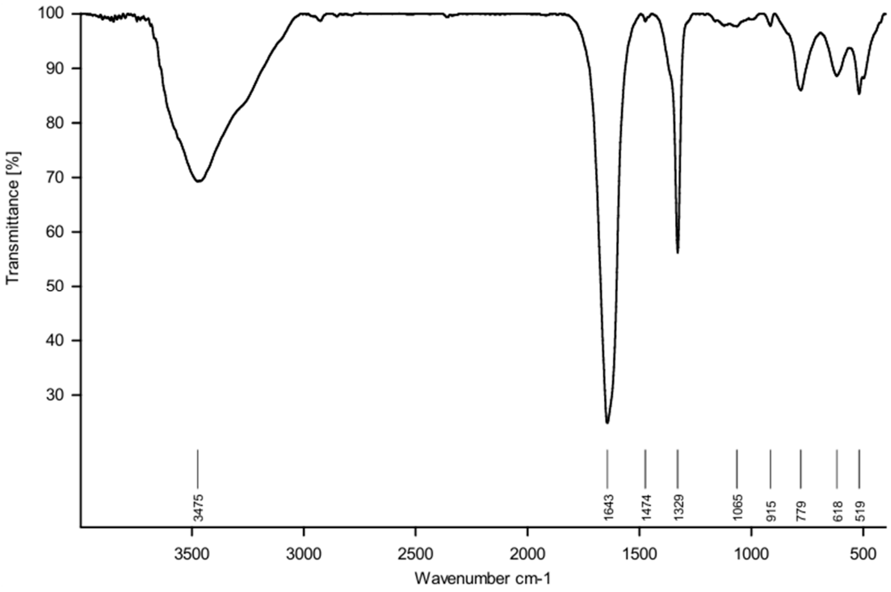

Figure S1.G FT-IR spectrum of the crystals isolated from the assay of crystallization of calcium oxalate in the presence of 2 mM citrate + 2 µM phytate, corresponding to calcium oxalate dihydrate (COD).

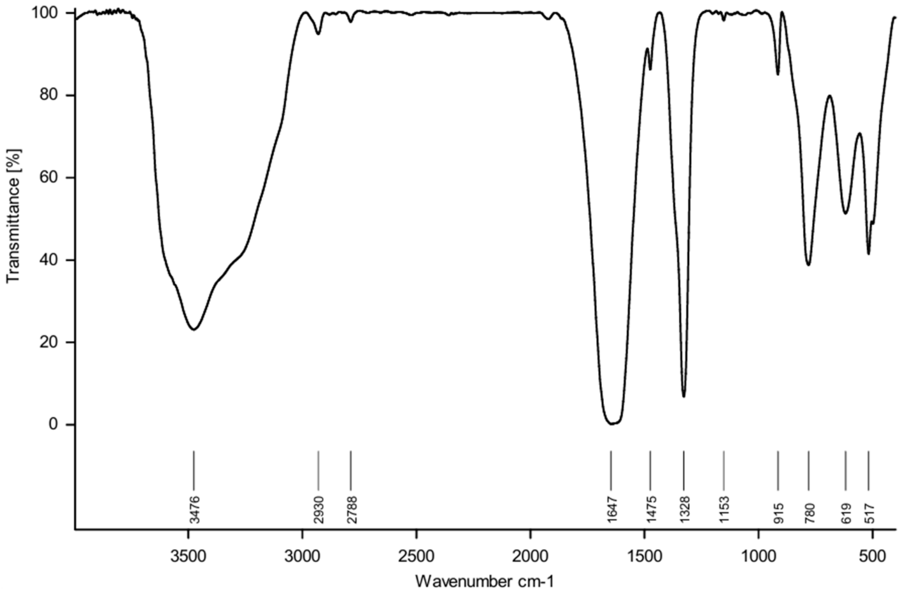

Figure S1.H FT-IR spectrum of the crystals isolated from the assay of crystallization of calcium oxalate in the presence of 2 mM tartronate + 2 µM phytate, corresponding to calcium oxalate dihydrate (COD).
